# Dynamics of Sulfate-Reducing Bacteria Community Structure in Surface Sediment of a Seasonally Hypoxic Enclosed Bay

**DOI:** 10.1264/jsme2.ME18092

**Published:** 2018-11-17

**Authors:** Fumiaki Mori, Yu Umezawa, Ryuji Kondo, Minoru Wada

**Affiliations:** 1 Graduate School of Fisheries and Environmental Sciences, Nagasaki University 852–8521, Nagasaki Japan; 2 Department of Marine Science and Technology, Fukui Prefectural University 917–0003, Fukui Japan

**Keywords:** enclosed bay, sediment, hypoxia, T-RFLP, sulfate-reducing bacteria

## Abstract

We herein report on the dynamics of a sulfate-reducing bacteria (SRB) community structure in the surface sediment of a seasonally hypoxic enclosed bay for two consecutive years (2012 and 2013). The uppermost (0–5 mm) and subsurface (5–10 mm) layers of sediment were examined with a terminal-restriction fragment length polymorphism (T-RFLP) analysis based on the dissimilatory sulfite reductase (*dsrA*) gene. The SRB community significantly differed between the two sediment layers over the sampling period. This difference was mainly attributed to operational taxonomic units (OTUs) that were unique to either of the sediment layers. However, nearly 70% of total OTUs were shared between the two layers, with a few predominating. Therefore, no significant shift was observed in the SRB community structure under varying dissolved oxygen (DO) conditions in bottom water overlying the sediment surface. An additional analysis of 16S rRNA gene amplicon sequences, conducted for three uppermost sediment samples (July, August, and September in 2012), revealed that *Desulfococcus*, a member of SRB with high tolerance to oxygen, was the predominant *Deltaproteobacteria* across the uppermost sediment samples. Based on the predominance of shared OTUs across the SRB community in the sediment (0–10 mm) regardless of bottom-water DO, some SRB that are physiologically tolerant of a wide range of DO conditions may have dominated and masked changes in responsive SRB to DO concentrations. These results suggest that the SRB community structure in the enclosed bay became stable under repeated cycles of seasonal hypoxia, but may be compromised if the severity of hypoxia increases in the future.

Sulfate-reducing bacteria (SRB) play key roles in the carbon and sulfur cycles of benthic environments through the dissimilatory sulfate reduction process (21). SRB mainly consume monomeric organic compounds and their fermented products, such as acetate, propionate, butyrate, and lactate (21), and are responsible for up to 50% of organic matter mineralization (13). As a result of the degradation of organic matter, SRB produce hydrogen sulfide (H_2_S), which accumulates in sediment, migrates upward to reach the overlying water, and consumes dissolved oxygen (DO) through abiotic and/or biotic processes. Therefore, SRB are largely responsible for oxygen dynamics in sediment and the water column under hypoxic conditions (8, 13, 26).

In fine-grained muddy sediments common to areas with high bacterial productivity, DO generally extends only a few millimeters or centimeters below the surface (9), leading to the formation of an oxic surface layer and permanently anoxic subsurface sediment layers. Even though SRB are abundant in the anoxic sediment layer, they are commonly found in oxic surface sediment layers (30). The presence of SRB in these oxic environments is often attributed to physiological mechanisms, such as superoxide dismutase, catalase, and NADH oxidase activities (30), through which SRB survive under otherwise lethal conditions. Since the extent of tolerance to O_2_ varies among SRB at the species or subspecies level (3, 30), the spatial gradient of DO may affect the distribution and composition of the SRB community in natural environments. At the order level, SRB belonging to *Desulfobacterales* and *Desulfarculales* were dominant in surface sediments at locations impacted by the hypoxic bottom-water mass, whereas these SRB groups were less abundant in oxic sediment (11, 18). At the genus level, SRB belonging to *Desulfococcus*, *Desulfovibrio*, and *Desulfonema* are often found in the oxic layer of microbial mats or aquatic sediments, whereas those inhabiting deeper, permanently anoxic sediment layers include *Desulfobulbus* and *Desulfobacter* (29, 30). These findings help infer the future status of coastal environments impacted by expanding hypoxia. However, since most of the studies conducted on spatial changes in SRB community structures in aquatic sediments were based on “snap-shot” sampling in the field, temporal variations in SRB community structures have not yet been extensively documented under changing bottom DO conditions. In order to obtain a more detailed understanding of the responses of the SRB community to spreading hypoxia in the coastal sea bottom, SRB communities need to be examined in a defined location that consistently experiences a wide range of DO conditions over many years.

The central region of Omura Bay, a seasonally hypoxic enclosed bay in Western Kyushu, is a natural laboratory in which a wide range of different DO conditions may be investigated due to consistent seasonal hypoxia, providing a suitable research environment to conduct the long-term monitoring of sediment bacterial communities (20, 22, 33, 35). During the hypoxic period (generally between July and September), the production and accumulation of H_2_S by SRB in the sediment becomes very active, contributing greatly to oxygen consumption by sediment (19), and apparently fueling the massive growth of *Beggiatoa*-like sulfur-oxidizing bacteria on surface sediment (37). Mori *et al.* (20) demonstrated that each of the uppermost (0–5 mm) and subsurface (5–10 mm) sediments harbored a distinctive bacterial community structure underlying hypoxic bottom water. They also showed that *Deltaproteobacteria*, presumably representing a large portion of the SRB community, increased in relative abundance as conditions became more hypoxic. However, the dynamics of the SRB community structure in Omura Bay remain largely unknown.

To fill this knowledge gap in the dynamics of the SRB community structure, we conducted a terminal restriction fragment length polymorphism (T-RFLP) analysis of dissimilatory sulfite reductase (*dsrA*) genes in the surface sediment of Omura Bay for two consecutive years (2012 and 2013). We also applied 454 pyrosequencing of the 16S rRNA gene in some selected samples from 2012 to gain deeper insights into the phylogenetic affiliation of the sediment SRB community. Based on the results obtained, we demonstrated the responses of the SRB community to DO availability in the bottom water of Omura Bay, and discussed the possible significance of these results in the context of global trends towards ocean deoxygenation.

## Materials and Methods

### Study site and sediment sampling

During the summer months in 2012 and 2013 (Table S1), sediment sampling was conducted at central Omura Bay (32°55.390N, 129°51.350E), as described by Mori *et al.* (20). In brief, three replicate sediment core samples were collected from the sampling station at a depth of 20±1 m with an acrylic pipe (31 cm in length and an inner diameter of 26 mm) by scuba diving, carefully avoiding areas with visible bioturbation. Three sediment cores were sectioned into subsamples for two layers: the uppermost layer (0–5 mm) and the subsurface layer (5–10 mm). Segments from the same depth were pooled and stored at −20°C until DNA extraction and total organic carbon (TOC) measurements. The DO, temperature, salinity, and chlorophyll fluorescence of vertical profiles in the sampling sites were obtained using a Conductivity Temperature Depth (CTD) profiler (AAQ; JFE-Advantec, Kobe, Japan). The TOC of sediment samples was measured according to the method of Wada *et al.* (36).

### T-RFLP analysis of *dsrA* genes

We used a T-RFLP analysis based on *dsrA* genes to reveal the SRB community structure. Approximately 1 g (wet weight) of thawed sediment was used for DNA extraction using an ISOIL DNA extraction kit (Nippon Gene, Tokyo, Japan), according to the protocols described by the manufacturer. Extracted DNA was then subjected to the T-RFLP assay described by Santillano *et al.* (28) with slight modifications. Briefly, extracted DNA was subjected to the PCR amplification of bacterial *dsrA* regions using the universal primer sets DSR1Fmix (containing DSR1F, DSR1Fa, DSR1Fb, DSR1Fc, and DSR1Fd) (17, 38, 41) and DSR1334R (28). PCR was performed in a 30-μL reaction mixture using PCR buffer, 15 U of KOD FX Neo DNA polymerase (Toyobo, Osaka, Japan), 12 nmol of dNTP mix, 6 pmol of each primer, and 30 ng of extracted DNA. The reaction was performed in a thermal cycler (DNA engine PTC-200; Bio-Rad, Hercules, CA, USA) using the following program: 1 cycle at 94°C for 2 min, followed by 30 cycles of 98°C for 10 s, 54°C for 30 s and 68°C for 60 s. The correct sizes of PCR products were verified by agarose gel electrophoresis (approximately 930 bp), and PCR products were further purified (QIAquick PCR purification kit; Qiagen, Hilden, Germany). After purification, approximately 400 ng of PCR products were digested in a 15-μL reaction volume with 20 U of *Nde*II (Nippon Gene) at 37°C for 2 h, followed by 15 min of heat inactivation at 65°C. Digested PCR products were then run on a 3730 DNA analyzer with the 1200LIZ marker (Applied Biosystems, Foster City, CA, USA), operating as a fragment analyzer. Electropherograms were then analyzed using Peak Scanner software (v1.0; Applied Biosystems), in which each peak displayed in an electropherogram represented one operational taxonomic unit (OTU) and the peak height represented the amount of the OTU present. Dynamic binning was performed for terminal restriction fragments (50–900 bp) as described by Ruan *et al.* (27). Maximum bin widths for the fragments 50–700 and 701–900 bp were 3 and 5 bp, respectively. The relative abundance of the respective OTUs to the community was estimated as peak height divided by the cumulative peak height of the given sample.

### Statistical analysis

T-RFLP data were primarily analyzed with PRIMER 6 (PRIMER-E, Plymouth, UK) (2). Similarities between samples were calculated using the Bray–Curtis algorithm. The resulting resemblance matrix was visualized using a non-parametric multidimensional scaling plot (nMDS). An analysis of similarities (ANOSIM) was performed on the Bray–Curtis resemblance matrix for *a priori* defined DO groups (oxic; >90 μM O_2_, dysoxic; 20–90 μM O_2_, and suboxic; <20 μM O_2_) and depth groups (the uppermost and subsurface layers). A similarity percentage analysis (SIMPER) was used to identify the OTUs that contributed the most to dissimilarities among the *a priori* defined groups (1). We also used the statistical program R (http://www.r-project.org/) for a heatmap analysis and backward stepwise multiple linear regression analyses to select the variables with the strongest ability to predict SRB richness (OTU numbers). Environmental data were logarithmically (log_10_+1) transformed before performing the multiple linear regression analysis. Outliers in the data set were detected using Grubbs’ outlier test at a 5% significance level before a multiple linear regression analysis. The Akaike information criterion, which balances the fit of a model against the number of parameters, was used to select the best fit model.

### Pyrosequencing analysis

Genomic samples were taken from the uppermost sediment layer in July, August, and September 2012, and were amplified for 454 sequencing using a forward and reverse fusion primer. The primers used were 27F (5′-AGA GTT TGA TCM TGG CTC AG-3′) and 519R (5′-GWA TTA CCG CGG CKG CTG-3′), both of which were designed for the bacterial V1–V3 hypervariable region of the 16S ribosomal RNA gene. PCR was conducted in 30 μL reaction mixture using PCR buffer, 0.75U of TaKaRa Ex Taq HS (Takara, Shiga, Japan), 12 nmol of dNTP mix, 5 pmol of each primer, and 20 ng of the extracted DNA. The program for 30 cycles of PCR had an initial step of 95°C for 5 min, followed by 95°C for 30 s, 58°C for 30 s, 72°C for 1 min, and finally 72°C for 5 min. PCR amplicons were then sent to Hokkaido System Science for DNA-tagged PCR and massively parallel pyrosequencing using a Roche 454 GS FLX Titanium System. In total, 11,748 sequences were generated and sorted.

After trimming, chimerical reads were detected by UCHIME (6). OTUs were clustered with a 97% similarity cut-off using UPARSE (7). Thereafter, respective OTUs were taxonomically assigned with the Greengenes “gg_13_8_99” reference taxonomy (http://www.mothur.org/wiki/Greengenes-formatted_databases) with Mothur (32). All associated raw data are available at the DDBJ Sequence Read Archive under accession numbers DRA006973 (DRX131732–DRX131734).

## Results and Discussion

**Fig. 1:**
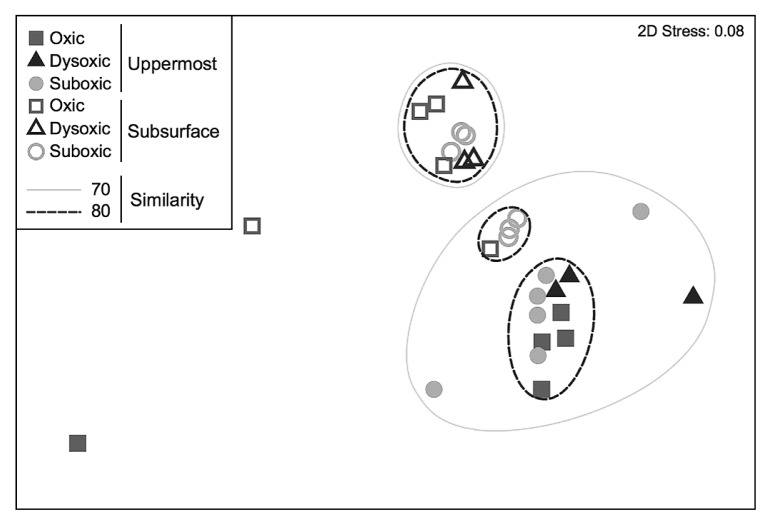
Non-metric multidimensional scaling ordination of a Bray–Curtis resemblance matrix of sulfate-reducing bacterial community assemblages calculated from terminal restriction fragment length polymorphisms for different oxygen conditions.

**Fig. 2:**
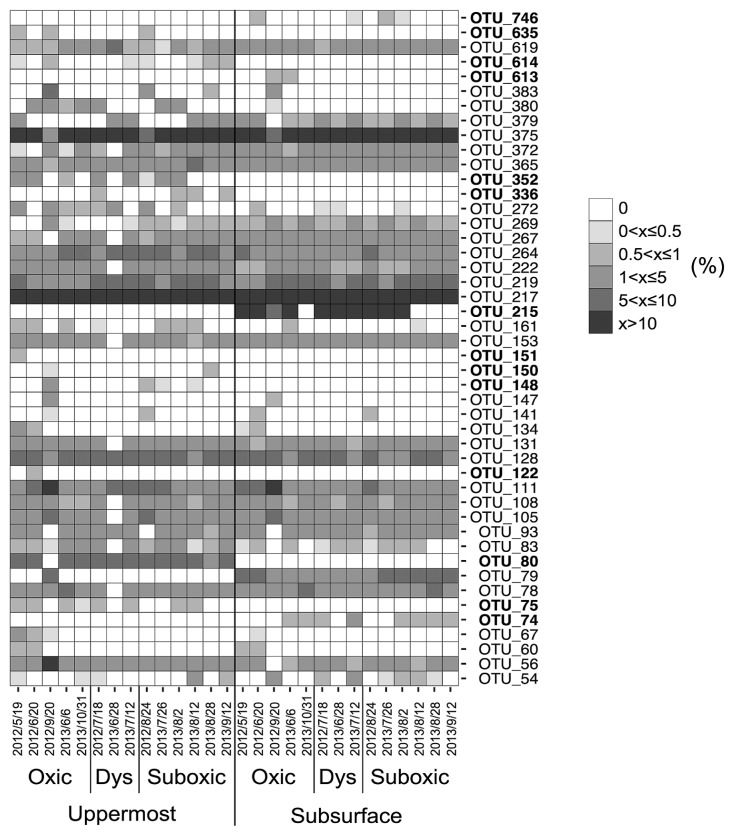
Heatmap of relative abundance of each *dsrA*-operational taxonomic unit (OTU) in uppermost and subsurface sediment layers. Colors represent the relative abundance of each OTU (%). The OTUs indicated by bold type were unique to either the uppermost or subsurface sediment layers. Oxygen conditions were divided into oxic (>90 μM O_2_), dysoxic (20–90 μM O_2_), and suboxic (<20 μM O_2_) conditions.

Forty-six distinctive OTUs of SRB were obtained throughout the present study, with 42 being identified in the uppermost layer and 36 in the subsurface layer. OTUs indicated that SRB communities significantly differed between the two sediment layers (ANOSIM, *R*=0.603, *P*<0.01) (Fig. 1 and 2, Table 1). Ten OTUs were unique in the uppermost layer (OTU_75, 80, 122, 148, 150, 151, 336, 352, 614, and 635) and 4 in the subsurface layer (OTU_74, 215, 613, and 746) (unique OTUs are represented in bold in Fig. 2). The SIMPER test revealed that OTU_215, which was unique and predominant in the subsurface layer, had the largest contribution (11.5%) to the dissimilarity between layers. Moreover, OTU_80, which showed the second largest contribution to the dissimilarity between layers (10.3%), was a unique OTU in the uppermost layer (Fig. 2, Table 2). These results indicate that SRB communities vertically differ within a depth of 1 cm. In addition, OTUs that are unique to either the uppermost or subsurface sediment layer contribute strongly to the vertical difference in the SRB community structure. Depth-related differences in the *dsrA* phylotypes in our study site suggest that biotic and/or abiotic conditions differed between the two sediment layers. One of the possible causes for depth-related differences is bioturbation mediated by burrow-building infauna. Mori *et al.* (20) found that small polychaetes generally formed a greater number of burrows in the uppermost layer than in the subsurface sediment layer during the normoxic period.

**Fig. 3:**
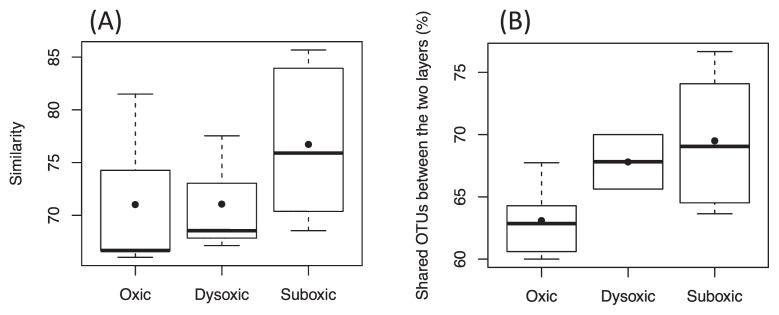
Box-and-whisker plots showing variations in relative ratios of shared OTUs between uppermost and subsurface sediment layers to the total number of detected OTUs on the same sampling day. The line inside the box indicates median values. Lines extending from the boxes represent minimum and maximum values. Closed circles represent means.

While the dissimilarity in the SRB community between the two sediment layers was largely attributed to the limited number of unique OTUs, nearly 70% of total OTUs were shared between the two layers, with a few predominating. OTU_217 and OTU_375 were dominant in both of the sediment layers across the different DO groups (Fig. 2, Fig. S1). As the proportion of shared OTUs slightly increased toward less oxic conditions (Fig. 3), similarities in the SRB community between the two sediment layers also slightly increased. Based on the predominance of shared OTUs among the SRB community in the two sediment layers regardless of bottom-water DO, the members of SRB that are physiologically tolerant of a wide range of DO conditions may have dominated and masked the influence of unshared and unique SRB that may otherwise lead to a major shift in the bacterial community due to changes in DO conditions. Consistent with this notion, the overall SRB community structure in the surface sediment (0–10 mm) was not clustered by the DO group (ANOSIM, *R*=−0.015, *P*=0.51 for the uppermost layer, *R*=−0.015, *P*=0.50 for the subsurface layer) (Fig. 1, Table 3).

**Fig. 4:**
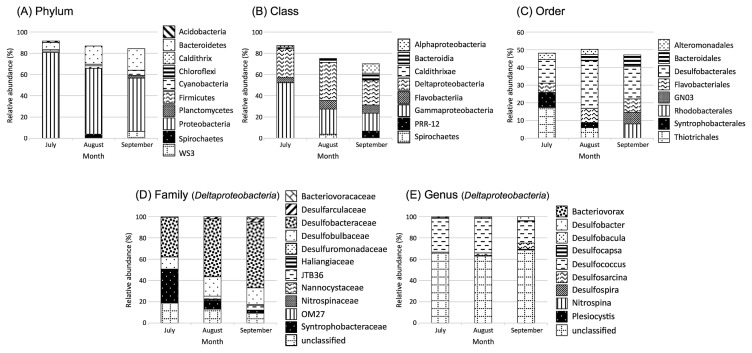
Bar plot of bacterial groups in pyrosequencing libraries of the uppermost sediment layer between June and September in 2012. At the Top, the bar plot shows the relative abundance of the five most abundant bacterial (A) phyla, (B) classes, and (C) orders. At the bottom, the bar plot shows the relative abundances of the bacterial (D) families and (E) genera within *Deltaproteobacteria* Phylum.

In addition, the present results indicate that the extent of the DO decline during summer (between July and September) for the two years (2012–2013) was not sufficiently low to cause significant shifts in the SRB community structure under bottom-water hypoxia. Further evidence cannot be provided because of the lack of sequence information from the results of the *dsrA*-TRFLP analysis. However, 16S rRNA gene tag sequencing for the uppermost sediment layers in July, August, and September 2012 provided information relevant to the above assumptions (Fig. 4). The sequences affiliated with *Deltaproteobacteria* were found to contribute more than one quarter of the total sequence reads across the sediment samples examined (between July and September, 2012). The sequence reads affiliated with the genus *Desulfococcus* within the family *Desulfobacteraceae* were predominant within *Deltaproteobacteria* in sediment samples, while more than 60% of sequences were not assigned to a taxon at the genus level (Fig. 4E). *Desulfococcus* was the most abundant under suboxic conditions and the least abundant under oxic conditions.

The inference derived from the *dsrA*-TRFLP analysis was supported further by the predominance of *Desulfococcus* in sediment samples. This SRB group is known to have the ability to tolerate oxic conditions and possibly to completely oxidize a number of organic substrates to CO_2_, which are common physiological features shared among almost all species belonging to the *Desulfococcus–Desulfonema* group (39). Isolated *Desulfococcus* have the capacity to survive under oxic conditions because of multiple defense mechanisms to molecular oxygen (5). Consistent with the results of 16S rRNA gene tag sequencing, the predominance of SRB affiliated with *Desulfobacteraceae* has been reported in other marine sediment surfaces *e.g.*, Jochum *et al.* [12]).

Abiotic factors other than DO (sediment TOC, bottom-water salinity and temperature) correlated with the richness of the SRB community. The temperatures of bottom water and TOC together explained 52% of the variance in SRB richness for the uppermost sediment layer, while the salinity of bottom water alone explained 42% of the variance for the subsurface sediment layer (Table 4). All of the parameters studied are known to affect the metabolic activities and, hence, distribution patterns of SRB (14–16, 24, 25, 31, 40). In contrast, bottom-water DO concentrations affected neither SRB richness nor the SRB community structure. This was again mainly due to the predominance of shared OTUs in the SRB community.

Due to the lack of *in situ* measurements of DO concentrations in sediment core samples in the present study, it may be difficult to directly argue an interaction between SRB and DO conditions within the sediment layers. However, as reported in the previous study, DO in the bottom water overlying the sediment surface may be used as a proxy for that within the surface sediment. This assumption may be valid at a depth layer of at least 0–5 mm in which bioturbation appears to be very active under normoxic conditions (20). Sediment below this depth must always be less oxic than that above. Although the validity of the proxy relationship between DO concentrations in interstitial water and that of overlying water in Omura Bay has not yet been elucidated in detail, the impact of bottom-water hypoxia on the dynamics of sediment bacterial community compositions (BCC) and activities in other field studies have been extensively reported (11, 18).

The present results are in contrast to our previous findings for the BCC of the same sediment samples with another molecular fingerprint technique, an automated ribosomal intergenic spacer analysis (ARISA) (20). Significant shifts in sediment BCC, as represented by the length polymorphism of the ribosomal intergenic spacer (rITS), were associated with varying DO availability in the bottom water of the study site (20). The contrasting response to DO concentrations between rITS and *dsrA* phylotypes may reflect differences in the extent of the phenotypic and phylogenetic diversities of bacteria that are represented by these genetic elements. BCC that are based on rITS may involve bacteria with greater diversities in the utilization of and sensitivities to DO than those based on *dsrA*.

Coverage and resolution achieved by T-RFLP are markedly lower than those of next generation sequencing (NGS) (4, 23, 34). Therefore, alpha diversity based on *dsrA*-TRFLP is more likely to markedly underrepresent the diversity of less abundant SRB members. According to the study by Hausmann *et al.* (10), less abundant (*i.e.*, “rare”) SRB members may respond to environmental perturbation more quickly than predominant SRB. However, this was not supported by the present results, which may have been because of the underrepresentation of SRB diversity by *dsrA*-TRFLP (Table S2). In contrast, T-RFLP is generally reported to provide β-diversity patterns that are similar to those obtained by NGS (4, 23, 34). The predominance of the two most abundant *dsrA*-TRFs (OTU_217 and OTU_375, Fig. S1) and the *Desulfococcus* reads of NGS data across the range of DO concentrations in the present study provides an example of congruence in diversity patterns between *dsrA*-TRFLP and NGS (Fig. 4).

In conclusion, SRB communities revealed by *dsrA*-TRFs significantly differed between the uppermost and subsurface sediment layers in Omura Bay, regardless of the changing concentrations of bottom-water DO that appeared to exert fundamental differences on the availability of DO, organic matter, and other electron acceptors across the sediment layers. Nearly 70% of total OTUs were shared between the two layers, with a few predominating in both. Based on the amplicon sequencing of the bacterial 16S rRNA gene, members of *Desulfococcus* (*Desulfobacteraceae*) represented the dominant population of SRB in surface sediment and appeared to be the strongest contributors to the stability of the bacterial community. However, a more prominent shift in the bacterial community structure may occur if the severity of hypoxia is exacerbated by the increases in water temperature associated with climate change. This may also compromise the stability of the sediment SRB community under seasonal hypoxia. Future research needs to involve the validation of the above-described inference in Omura Bay in order to better predict the possible consequences imposed by global trends in ocean deoxygenation.

## Supplementary Material



## Figures and Tables

**Table 1:** Analysis of similarity (ANOSIM) between uppermost and subsurface sediment layers in different oxygen groups.

Oxic		Dysoxic		Suboxic		All	
			
*R*	*P*	*R*	*P*	*R*	*P*	*R*	*P*
**0.372**	**0.02**	0.815	0.1	**0.617**	**0.002**	**0.603**	**0.001**

Bold indicates a significant value at the *P*<0.05 level.

**Table 2:** Summary of major *dsrA*-operational taxonomic units (OTUs) contributing up to approximately 50% cumulative dissimilarity between uppermost and subsurface layers.

Rank	OTU	Dissimilarity contributions (%)	Cumulative contribution (%)
1	215*	11.5	11.5
2	80*	10.3	21.8
3	79	9.5	31.3
4	217	3.1	34.4
5	375	2.9	37.3
6	379	2.8	40.1
7	352*	2.6	42.7
8	272	2.6	45.3
9	269	2.4	47.7
10	54	2.4	50.1

* indicates unique OTUs of each sediment layer

**Table 3:** Analysis of similarity (ANOSIM) between oxic, dysoxic, and suboxic oxygen groups in uppermost and subsurface sediment layers.

	Oxic vs Dysoxic		Oxic vs Suboxic		Dysoxic vs Suboxic		All	
			
	*R*	*P*	*R*	*P*	*R*	*P*	*R*	*P*
Uppermost	0.067	0.286	−0.045	0.654	−0.043	0.536	−0.015	0.513
Subsurface	−0.138	0.768	0.008	0.169	−0.056	0.548	−0.015	0.500

**Table 4:** Multiple linear regression using abiotic parameters to explain sulfate-reducing bacterial richness and diversity

	Explanatory variable	Richness		

		Regression coefficient	*P*	Model statistics
Uppermost	DO^a^	—	—	*R*^2^=0.60
	Temp	−18.0	<0.05	*R*^2^_adj._=0.52
	Salinity	—	—	*P*<0.05
	TOC^b^	−22.4	<0.01	
	Intercept	85.8	<0.01	

Subsurface	DO^a^	—	—	*R*^2^=0.47
	Temp	—	—	*R*^2^_adj._=0.42
	Salinity	73.2	<0.01	*P*<0.01
	TOC^b^	—	—	
	Intercept	−86.0	<0.05	

a dissolved oxygen

b total organic carbon
