# Supplementary material for: Dynamics of Sulfate-Reducing Bacteria Community Structure in Surface Sediment of a Seasonally Hypoxic Enclosed Bay
Source: Microbes Environ. 2018 Nov 17;33(4):378–84. doi: 10.1264/jsme2.ME18092 (PMC6308007; doi:10.1264/jsme2.ME18092)
Supplement: Supplementary file 1 [file 33_378_s1.pdf]

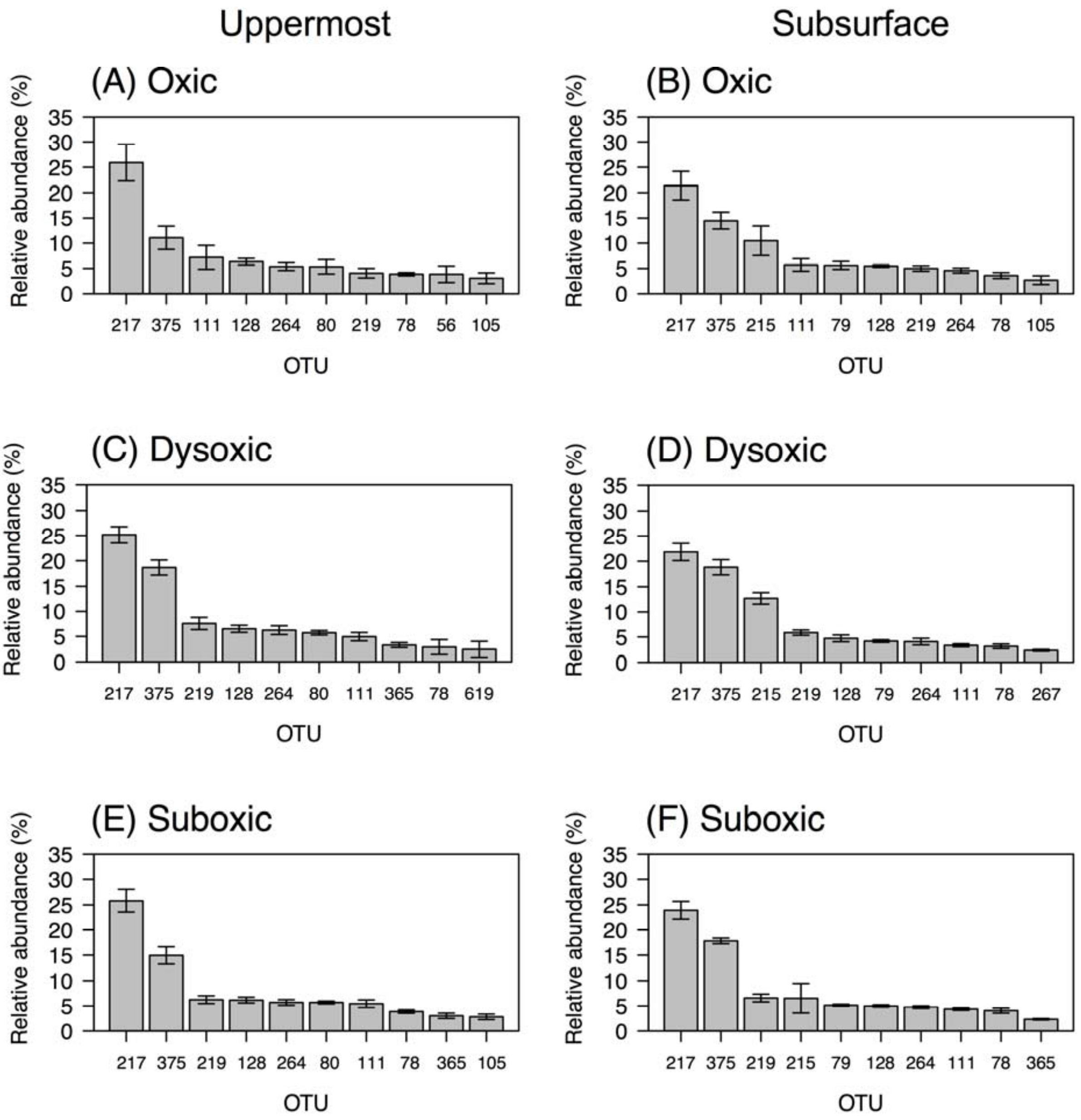

Fig. S1 Mean rank abundance plots of the *dsrA*-terminal restriction fragment for the top 10 operational taxonomic units (OTUs). The *y* axis shows the means of relative abundance of each OTU within same oxygen groups, whereas the *x* axis is the ordinal rank of OTUs from most abundant to least abundant. Error bars represent standard errors (n = 6 for oxic samples, 3 for dysoxic samples, and 6 for suboxic samples). .

Table S1 Environmental parameters and diversity indices for sulfate reducing bacteria (SRB) communities in Omura Bay

Year	Month/Day	Overlying water				Uppermost sediment			Subsurface sediment			Shared OTU[a]
		DO conditions	DO (μM)	Temperature (°C)	Salinity (PSU)	OTU[a]	Shannon (H')	TOC[b] (mg g-1)	OTU	Shannon (H')	TOC[b] (mg g-1)	
2012	5/19	Oxic	157.6	15.7	32.9	32	2.78	31.3	25	2.64	36.3	22
	6/20	Oxic	124.0	19.0	33.1	27	2.63	32.2	29	2.74	35.5	21
	7/18	Dysoxic	82.7	22.1	31.9	27	2.62	33.1	26	2.66	34.2	21
	8/24	Suboxic	0.0	24.1	31.3	27	2.77	30.6	24	2.66	36.4	20
	9/20	Oxic	195.9	27.3	31.2	29	2.89	22.0	24	2.85	39.7	20
2013	6/6	Oxic	138.2	17.6	32.9	26	2.52	37.8	26	2.64	39.6	21
	6/28	Dysoxic	70.7	20.8	31.3	13	2.18	36.5	24	2.53	38	12
	7/12	Dysoxic	53.2	22.7	32.8	25	2.65	39.5	26	2.60	35.9	21
	7/26	Suboxic	1.6	22.3	30.4	25	2.60	37.4	25	2.63	38.6	20
	8/2	Suboxic	1.8	22.8	32.7	27	2.67	38.3	27	2.64	38.6	21
	8/12	Suboxic	8.0	24.6	32.6	28	2.72	35.3	25	2.59	37.9	23
	8/28	Suboxic	3.1	26.4	32.7	25	2.55	31.8	23	2.49	37.8	20
	9/12	Suboxic	2.9	26.9	28.5	25	2.52	36.4	22	2.47	35.9	20
	10/31	Oxic	166.7	22.1	30.6	24	2.50	37.0	22	2.41	38.8	18
Average	n = 5	Oxic	156.5	20.3	32.1	27.6	2.7	32.1	25.2	2.7	38.0	20.4
	n = 3	Dysoxic	68.8	21.8	32.0	21.7	2.5	36.4	25.3	2.6	36.0	18
	n = 6	Suboxic	2.9	24.5	31.4	26.2	2.6	34.9	24.3	2.6	37.5	20.7
	n = 14	All samples	71.9	22.4	31.8	25.7	2.6	34.2	24.9	2.6	37.4	20.0

a: Operational taxonomic unit

b: Total organic carbon

Table S2 Mean rank abundance of the dsrA-operational taxonomic units (OTUs) within same oxygen groups.

(A) Uppermost sediment layer

Rank (All sample)	OTU	Uppermost: Oxic Relative abundance	SD	Dysoxic Relative abundance	SD	Suboxic Relative abundance	SD	All samples Relative abundance	SD
1	217	25.91	8.03	25.16	2.76	25.80	5.41	25.70	5.69
2	**375**	11.07	5.13	18.64	2.61	14.95	4.17	14.35	4.94
3	128	6.34	1.46	6.50	1.21	6.15	1.40	6.30	1.28
4	111	7.19	5.29	4.96	1.42	5.44	1.85	5.96	3.34
5	**219**	4.06	2.10	7.55	2.08	6.23	1.89	5.74	2.31
6	**264**	5.36	1.72	6.24	1.50	5.69	1.36	5.69	1.44
7	**80**	5.32	3.18	5.72	0.75	5.69	0.69	5.56	1.85
8	**78**	3.87	0.70	2.92	2.53	3.88	0.81	3.67	1.25
9	**365**	2.24	0.85	3.31	0.84	3.03	1.28	2.81	1.08
10	105	3.07	2.37	1.54	1.35	2.82	1.33	2.64	1.75
11	56	3.85	3.66	1.77	0.04	1.99	0.65	2.61	2.28
12	222	2.22	1.43	1.49	1.61	2.04	0.58	1.99	1.11
13	**131**	2.08	0.36	1.50	1.31	2.12	0.28	1.97	0.63
14	**153**	1.73	0.33	1.44	1.25	1.73	0.54	1.67	0.63
15	**619**	0.92	0.26	2.48	2.71	1.06	0.50	1.32	1.29
16	**372**	1.17	1.61	0.70	0.62	1.58	0.73	1.25	1.09
17	93	1.05	0.61	1.31	0.27	0.99	0.51	1.08	0.49
18	**83**	1.10	0.56	0.87	0.76	1.13	0.46	1.06	0.53
19	**108**	1.06	0.09	0.78	0.68	1.13	0.13	1.03	0.32
20	**267**	0.90	0.53	0.87	0.75	1.21	0.22	1.03	0.47
21	383	1.17	2.61	0.00	0.00	0.53	1.00	0.64	1.64
22	272	0.82	0.54	0.93	0.93	0.32	0.51	0.63	0.63
23	269	0.79	1.59	0.10	0.18	0.76	0.17	0.63	0.94
24	352	0.64	0.59	0.85	0.74	0.44	0.57	0.60	0.59
25	380	0.89	0.53	0.43	0.74	0.36	0.56	0.56	0.60
26	**379**	0.20	0.46	0.87	0.81	0.66	0.73	0.54	0.67
27	79	1.15	2.58	0.00	0.00	0.00	0.00	0.41	1.54
28	75	0.53	0.37	0.52	0.47	0.25	0.39	0.41	0.39
29	161	0.38	0.36	0.10	0.18	0.37	0.40	0.32	0.35
30	**614**	0.26	0.39	0.10	0.18	0.34	0.31	0.26	0.31
31	148	0.31	0.70	0.00	0.00	0.25	0.34	0.22	0.46
32	**54**	0.13	0.18	0.10	0.18	0.32	0.54	0.21	0.37
33	**336**	0.00	0.00	0.25	0.43	0.32	0.35	0.19	0.31
34	635	0.32	0.44	0.00	0.00	0.15	0.38	0.18	0.36
35	67	0.45	0.48	0.00	0.00	0.00	0.00	0.16	0.35
36	60	0.37	0.51	0.00	0.00	0.00	0.00	0.13	0.34
37	134	0.36	0.51	0.00	0.00	0.00	0.00	0.13	0.33
38	147	0.24	0.55	0.00	0.00	0.00	0.00	0.09	0.33
39	**141**	0.09	0.19	0.00	0.00	0.12	0.30	0.08	0.22
40	**150**	0.08	0.17	0.00	0.00	0.11	0.27	0.07	0.20
41	151	0.16	0.35	0.00	0.00	0.00	0.00	0.06	0.21
42	122	0.15	0.33	0.00	0.00	0.00	0.00	0.05	0.20

Bold indicates more abundant OTUs in suboxic condition as compared to that in oxic condition.

(B) Subsurface sediment layer

Rank (All sample)	OTU	Subsurface: Oxic Relative abundance	SD	Dysoxic Relative abundance	SD	Suboxic Relative abundance	SD	All samples Relative abundance	SD
1	217	21.38	6.29	21.86	3.00	23.95	4.30	22.58	4.71
2	375	14.48	3.77	18.80	2.64	17.81	1.35	16.83	3.10
3	215	10.48	6.52	12.69	1.98	6.51	7.17	9.25	6.34
4	219	4.99	1.19	5.85	0.89	6.58	1.87	5.85	1.56
5	79	5.60	1.77	4.18	0.44	5.13	0.51	5.09	1.17
6	128	5.46	0.53	4.72	1.17	4.97	0.59	5.09	0.72
7	111	5.69	2.75	3.35	0.51	4.37	0.63	4.62	1.83
8	264	4.58	1.10	4.09	1.13	4.76	0.63	4.55	0.89
9	78	3.59	1.30	3.17	0.77	4.06	1.22	3.70	1.15
10	365	2.08	0.58	2.06	0.17	2.34	0.35	2.18	0.42
11	105	2.68	1.93	1.22	0.15	1.79	0.47	1.99	1.26
12	267	1.51	0.40	2.37	0.32	1.89	0.26	1.86	0.45
13	131	1.66	0.86	1.28	0.30	2.13	0.66	1.78	0.73
14	153	1.52	0.32	2.04	0.36	1.70	0.32	1.71	0.36
15	619	1.59	0.21	1.48	0.42	1.53	0.24	1.54	0.26
16	56	1.26	0.94	1.69	0.97	1.56	0.37	1.48	0.71
17	372	1.31	0.51	1.80	0.45	1.33	0.29	1.42	0.43
18	222	1.61	1.09	0.85	0.23	1.34	0.52	1.33	0.75
19	108	1.42	0.58	1.20	0.27	1.21	0.28	1.28	0.39
20	93	1.06	0.63	1.17	0.18	1.39	0.30	1.22	0.43
21	269	1.13	0.48	0.79	0.27	0.89	0.13	0.95	0.33
22	379	0.76	0.49	1.06	0.14	0.99	0.11	0.92	0.31
23	54	0.99	2.21	0.67	0.64	0.39	0.38	0.66	1.31
24	83	0.38	0.41	0.69	0.35	0.44	0.40	0.47	0.38
25	74	0.27	0.37	0.61	0.61	0.55	0.44	0.46	0.44
26	383	0.91	2.03	0.00	0.00	0.00	0.00	0.32	1.21
27	746	0.12	0.27	0.11	0.18	0.17	0.30	0.14	0.25
28	272	0.17	0.38	0.20	0.17	0.05	0.13	0.13	0.25
29	60	0.31	0.42	0.00	0.00	0.00	0.00	0.11	0.28
30	141	0.15	0.34	0.00	0.00	0.13	0.31	0.11	0.28
31	613	0.29	0.40	0.00	0.00	0.00	0.00	0.10	0.27
32	161	0.12	0.28	0.00	0.00	0.05	0.13	0.07	0.18
33	134	0.18	0.27	0.00	0.00	0.00	0.00	0.07	0.18
34	147	0.14	0.32	0.00	0.00	0.00	0.00	0.05	0.19
35	67	0.08	0.18	0.00	0.00	0.00	0.00	0.03	0.11
36	380	0.07	0.15	0.00	0.00	0.00	0.00	0.02	0.09

Bold indicates more abundant OTUs in suboxic condition as compared to that in oxic condition.
